# Elevated angiopoietin 2 in aqueous of patients with neovascular age related macular degeneration correlates with disease severity at presentation

**DOI:** 10.1038/srep45081

**Published:** 2017-03-27

**Authors:** Danny S. Ng, Yolanda W. Yip, Malini Bakthavatsalam, Li J. Chen, Tse K. Ng, Timothy Y. Lai, Calvin P. Pang, Mårten E. Brelén

**Affiliations:** 1 Department of Ophthalmology and Visual Sciences The Chinese University of Hong Kong Hong Kong Eye Hospital, 147K Argyle Street, Hong Kong.

## Abstract

Angiopoietin 2 (ANG2) is a proangiogenic cytokine which may have an implication in neovascular age related macular degeneration (nAMD). In 24 eyes of 24 subjects presenting with treatment naïve nAMD and 26 eyes of 26 control patients, aqueous humor samples were collected at the time of intervention (intravitreal injection of anti-vascular endothelial growth factor or cataract extraction). Best corrected visual acuity (BCVA) with and central macular thickness (CMT) using optical coherence tomography (OCT) were measured before each injection in the nAMD group. Aqueous cytokine levels were determined by immunoassay using a multiplex array (Quansys Biosciences, Logan, UT). Levels of ANG2 in the aqueous were significantly higher in nAMD patients than those of the control group (p < 0.0001), so were hepatocyte growth factor (HGF), interleukin-8 (IL-8) and tissue inhibitor of metalloproteinase 1 (TIMP 1), all with p < 0.001. ANG2 correlated with worse BCVA (r = 0.44, p-value = 0.027) and greater CMT (r = 0.66, p-value < 0.0001) on optical coherence tomography (OCT). ANG2 is upregulated in patients with nAMD and correlates with severity of disease at presentation.

The development of neovascular age related macular degeneration (nAMD) is mediated by a variety of factors including the upregulation of oxidative stress, the activation of pro-inflammatory and angiogenic cytokines and, most importantly, the high expression of vascular endothelial growth factor (VEGF). Blocking VEGF with monoclonal antibodies (Bevacizumab; Roche Pharmaceuticals), antibody fragments (Ranibizumab; Roche Pharmaceuticals) or fusion proteins (Aflibercept, Bayer Pharmaceuticals) has already demonstrated in large multicentre clinical trials to be effective in preventing sight loss due to nAMD^1^,^2^,^3^. There are however still a proportion of patients who do not respond to conventional therapy. In various retrospective studies the reported rate of non-responders is between 10–15%^4^,^5^, regardless of whether Bevacizumab or Ranibizumab monotherapy was being used^6^. One possible explanation for the high rate of non-responders is that anti-VEGF monotherapy only blocks one pathway of pathological angiogenesis and other angiogenic factors may be responsible for the disease progression. Certainly, many reports have suggested that switching patients to Aflibercept which blocks multiple angiogenic growth factors including VEGF-A, VEGF-B and PIGF (placental growth factor) have better treatment outcomes than anti-VEGF monotherapy^7^,^8^. In addition, combination therapy with anti-VEGF and corticosteroid has better treatment outcomes than anti-VEGF monotherapy^9^,^10^. There is currently great research interest in discovering new angiogenic pathways that may serve as therapeutic targets to achieve better outcomes in the treatment of nAMD in the future.

Various cytokines have been implicated in the pathogenesis of cancers, a disease process not dissimilar to nAMD^11^. Indeed, bevacizumab, which was originally developed to treat colon cancer, has proven extremely effective in treating nAMD when injected intravitreally^2^. Angiopoietin 2 (ANG2) is a proangiogenic cytokine which binds to the Tie2 receptor on endothelial cells in blood vessels^12^. Neutralizing molecules to ANG2 can block tumor growth *in vitro*^13^, which subsequently led to the use of anti-ANG2 monoclonal antibodies in clinical trials for the treatment of solid tumors^14^,^15^. ANG2 has previously been implicated in nAMD but, to our knowledge, the intraocular levels of ANG2 in patients with treatment naïve nAMD have not been previously investigated. This study therefore aimed to explore the correlation of ANG2 concentration in aqueous humor (together with other previously implicated angiogenic cytokines) with disease severity of nAMD at presentation.

## Methods

Patients were recruited from The Chinese University of Hong Kong Eye Centre and The Hong Kong Eye Hospital. This study followed the tenets of the Declaration of Helsinki and is approved by the Kowloon Central Cluster/Kowloon East Cluster Research Ethics Institutional Review Board. Written informed consent was obtained from all patients before enrollment.

### Subjects

Aqueous samples were taken from consecutive 24 eyes of 24 patients with treatment naïve nAMD at first presentation. Aqueous was also collected from consecutive 26 age matched control subjects during cataract surgery in patients with no clinical signs of AMD or other significant eye pathology. The demographics of the patients, systemic medical history as well as the subtype of nAMD diagnosed on fluorescein angiography and indocyanine green angiography is shown in Table 1.

The inclusion criteria for nAMD patients were the presence of active choroidal neovascular membranes (CNV). All subtypes of CNV were included in this study (see Table 1). Patients were only included if they had received no previous intravitreal injections of anti-VEGF (i.e. treatment naïve). Patients were excluded if they had retinal disease other than nAMD. In particular, patients with diabetic retinopathy were excluded from the study. Patients with raised intraocular pressure above 21 mmHg were also excluded. In the control group, patients were included if they had no ocular pathology except the presence of cataract. Again, diabetic patients were excluded from the control group.

### Clinical assessment

All patients underwent best corrected visual acuity assessment (BCVA), biomicroscopic slit lamp examination including dilated fundus examination and intra-ocular pressure measurement by applanation tonometry. All patients had a macular scan with optical coherence tomography (Stratus, Carl Zeiss Meditec) from which the central macular thickness (CMT) was measured. The nAMD patients also underwent fluorescein angiography and indocyanine green angiography in order to confirm the diagnosis of nAMD and to subtype the choroidal neovascular membrane.

### Aqueous collection procedure

In the nAMD group the aqueous samples were collected immediately before performing an intravitreal injection of anti-VEGF. After the eyelids and conjunctiva were disinfected with povidine iodine a sterile drape was placed and an eyelid speculum inserted. The aqueous was collected using a 1 ml syringe with attached 30 G needle. In the control group the aqueous was sampled after povidine iodine, sterile drape and eyelid speculum was inserted but before the cataract procedure was performed. A paracentesis was made with a 15° blade and the aqueous sampled with a 1 ml syringe with attached 30 G needle. In both groups the collected aqueous samples were immediately centrifuged at 1500 rpm for 5 min at 4 °C and stored at −80 °C.

### Cytokine analysis of aqueous humor

A multiplex ELISA array (Quansys Biosciences, Logan, UT) was used to quantify the levels of 9 human angiogenic factors (Vascular Endothelial Growth Factor: VEGF, Angiopoietin 2: ANG2, basic Fibroblast Growth Factor: bFGF, Hepatocyte Growth Factor: HGF, Platelet Derived Growth Factor: PDGF, Interleukin 8: IL8, Tissue Inhibitor of MetalloProteinases 1 and 2: TIMP1, and TIMP2 and Tumour Necrosis Factor alpha: TNFα) in all aqueous samples. In brief, total protein level of all aqueous samples was determined by the total protein assay (Biorad). Equal amount of total protein (1 μg) for each aqueous samples was applied to the well (duplicated wells for each sample), and incubated at room temperature for 1 hour. After washing with the Wash Buffer 3 times, Detection Mix was added to the plate and incubated at room temperature for 1 hour. After washing 3 times, horse radish peroxidase-conjugated Streptavidin was applied and incubated at room temperature for 15 minutes. After washing 6 times, substrate was added and the plate was imaged by Chemi-Doc (Biorad). The intensities of the spots were quantified and calculated by the Q-View software (Quansys Biosciences) according to the standard curves for each factor. Mean of the duplicated samples was considered as the data point of each sample.

### Statistical analysis

The statistical analysis was performed using commercially available software (SPSS, Chicago, IL, USA). Chi square test was used for categorical analysis. Non-parametric Mann-Whitney U test was used to calculate statistical significance of cytokine levels in nAMD patient versus controls. Spearmann correlations were used to correlate cytokine levels with clinical outcomes of BCVA and CMT on OCT.

## Results

The study and control subjects were similar in age, and there were no statistically significance differences in the number of subjects with hypertension, hyperlipidemia and ischemic heart disease. The mean +/− standard deviation (SD) CMT of the nAMD eyes was significantly higher (353 +/− 140.34 μm vs. 229.05 +/− 26.99 μm, p = <0.001). The mean levels of ANG2 in the nAMD group (57.39 +/− 68.78 pmol/ml) was significantly higher (p < 0.001) than in the control group (10.46 +/− 5.90 pg/ml). A much broader range of ANG2 levels were found in nAMD patients whereas in control subjects all patients had ANG2 levels which were near the minimum detectable level. ANG2 levels did not correlate with patient demographics such as age or gender. Other growth factors including hepatocyte growth factor (HGF), Interleukin-8 (IL8) and tissue inhibitor of metalloproteinase 1 (TIMP1) were also higher in the nAMD group compared to controls (p < 0.001). Notably, the levels of VEGF were not significantly higher in the nAMD group as compared to controls (p = 0.085). The levels of growth factors in nAMD and controls are shown in Table 2.

**Figure 1:**
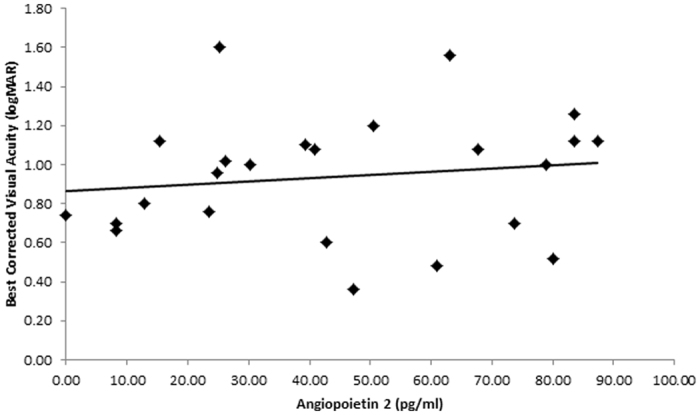
Correlation of Angiopoietin 2 levels (pg/ml) with best corrected visual acuity (logMAR) in neoavascular AMD patients. The correlation coefficient r = 0.44 and p-value = 0.028.

The levels of ANG2 had a positive correlation with BCVA (r = 0.44, p-value = 0.028). Namely, a higher ANG2 level was associated with a worse BCVA at presentation (Fig. 1). This correlation was not found with any of the other growth factors analysed in this study.

**Figure 2:**
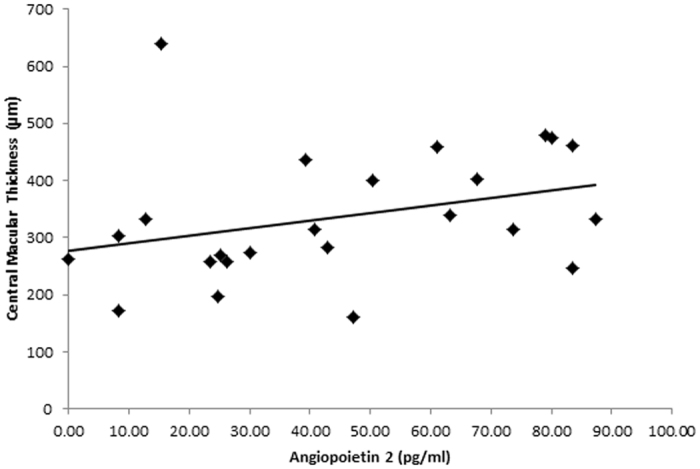
Correlation of Angiopoietin 2 levels (pg/ml) with central macular thickness (μm) on optical coherence tomography (OCT) scan. The correlation coefficient r = 0.66 and p-value < 0.0001.

ANG2 was also strongly correlated with CMT on OCT scan (r = 0.66, p-value < 0.0001). Namely, a higher ANG2 level was associated with a greater CMT (Fig. 2).

The correlation coefficients and respective P-values of all cytokines with BCVA and CMT on OCT are shown in Table 3. Only ANG2 showed a positive correlation to both BCVA (r = 0.44 and p = 0.028) and CMT (r = 0.66 and p-value < 0.0001). An association was also found between bFGF levels and CMT (r = 0.49 and p = 0.012).

## Discussion

The results from our study showed that ANG2 was higher in the aqueous of nAMD patients as compared to control subjects (p < 0.0001). This contradicts previous published reports showing lower ANG2 in aqueous of nAMD patients^16^. One possibility for the difference in results is that we analysed the ANG2 levels individually for each patient rather than mixing the aqueous of all samples and performing a single assay as reported by Sung HJ *et al*.^16^. Our results show a broad range of ANG2 levels in the aqueous of patients with nAMD and mixing samples may lead to differences in aqueous levels becoming statistically insignificant. Other studies have shown only a marginal increase in ANG2 in the vitreous (as opposed to aqueous) of patients with nAMD versus controls (p = 0.049)^17^. Our results also confirmed the upregulation of HGF which has been previously demonstrated in an animal model of CNV^18^ and in aqueous of patients with nAMD^19^. The same study by Jonas *et al*. also showed elevated levels of IL8 in patients with nAMD which was a result reproduced in our study^19^. TIMP1 has also previously been reported to have higher intraocular levels in nAMD patients than controls^20^.

Contrary to previous reports which demonstrated increased aqueous VEGF levels in nAMD eyes^21^,^22^,^23^,^24^, this study did not find significantly elevated VEGF concentrations in the aqueous of nAMD patients compared to control patients. A number of previous studies of nAMD eyes also did not detect significant differences in aqueous VEGF concentrations between treatment naïve, recurrent CNV and control groups^19^,^25^,^26^. Dilution of aqueous samples was required in ELISA techniques, which could have reduced the sensitivity for detecting VEGF. Another plausibility which could influence the sensitivity for measuring VEGF levels was insufficient volume of aqueous humor samples obtained from patients. Ultrasensitive methods, such as the paper-based ELISA, are being developed for the measurements of aqueous cytokine levels from small clinical samples and further studies would validate the reliability of new techniques^27^.

This is the first study, to our knowledge, that has shown ANG2 to be elevated in aqueous of patients with nAMD. The effect of ANG2 in the pathogenesis of neovascularization is complex and context dependent. ANG2 competes with ANG1 for binding on the Tie2 receptor which is expressed on endothelial cells of blood vessel walls. In the presence of high VEGF levels, ANG2/Tie2 is pro-angiogenic leading to loss of endothelial cell junction integrity, pericyte drop-off and angiogenic sprouting^28^. It also upregulates inflammation by making the endothelial cells more sensitive to the effects of TNFα^29^. Thus, ANG2 appears to be a common signaling pathway for both angiogenesis and inflammation^30^. However, in the absence of VEGF, ANG2 is anti-angiogenic by inducing endothelial cell apoptosis and regression of neovascularisation. While Ang1 is produced by periendothelial cells, ANG 2 is stored in Weibel-Palate bodies within endothelial cells and can therefore be rapidly released^29^,^31^. This makes ANG2 a more attractive drug target than Ang 1 for inhibiting angiogenesis^12^.

Angiopoietins have previously been implicated in the pathogenesis of nAMD both in animal and clinical studies. In an animal model of laser-induced CNV, intravitreal administration of ANG1 prevented CNV formation and suppressed vascular leakage. The authors attributed this effect of ANG1 to the inhibition of macrophages which secrete VEGF and the tightening of endothelial cell junctions^32^. High expression of ANG2 has been shown to augment vascular permeability from VEGF. *In vitro* studies using retinal endothelial cells have shown ANG2 to increase permeability by 25–30% in the absence of VEGF, but in the presence of VEGF the permeability increases 100–400%^33^. In previous clinical studies, two publications have shown that VEGF and ANG2 are expressed in surgically excised CNV in patients with nAMD^34^,^35^. In addition, within the CNV lesion, ANG2 expression was higher than ANG1 and was co-localised to areas of VEGF expression^36^.

It remains unclear whether the elevated levels of ANG2 in aqueous humor may be a consequence of a breakdown in the blood retinal barrier or if raised levels are the result of hyper-production leading to nAMD^19^. Furthermore, we are uncertain about the relationship between systemic levels of ANG2 with its aqueous concentration. The sample size in this study was too small for a meaningful multivariate analysis, so it remains inconclusive whether inter-dependencies between the various cytokines exist. Nevertheless, all of the nAMD subjects in this study were part of a larger cohort in which we have identified the association of ANG2 gene in our population. (Ma L and Chen L. J. *et al*., manuscript in press). The result from our study may serve to further explore this by, for example, performing immunohistochemical studies on the retina of CNV induced animal models. Other animal models of nAMD have shown that combined blockage of VEGF and upregulation of ANG1/TIE2 (through the inhibition of vascular endothelial-protein tyrosine phosphatase, VE-PTP) greatly suppresses neovascularisaton^37^. The same VE-PTP inhibitor has subsequently been used in patients with diabetic macular edema showing no safety concerns and a greater efficacy with combination therapy (VE-PTP + anti-VEGF)^38^. Clinical studies are currently ongoing investigating the effect of combined VEGF and VE-PTP inhibition in patients with macular edema secondary to retinal vein occlusions. Based on the results of this study, which show elevated levels of ANG2 in patients with nAMD, it may be possible that combined VEGF and VE-PTP blockage can produce better treatment outcomes in this condition as well. We have not recruited subjects with dry AMD undergoing cataract extraction surgery for aqueous analysis which would provide further insights into the aqueous cytokines profile in this cohort.

In conclusion, the role of ANG2 in angiogenesis and inflammation has been elucidated in molecular studies in animal models^11^,^39^,^40^. Its safety and efficacy as a therapeutic targeted is being determined in ongoing clinical trials^12^. Despite our relatively small sample size, we have demonstrated increased levels of ANG2 in the aqueous humor of nAMD eyes, as well as its association with BCVA and CMT at presentation. An ongoing longitudinal study will find out whether ANG2 levels at presentation are predictive of treatment outcomes and could therefore become a prognosticating marker and how the levels of ANG2 alter with anti-VEGF monotherapy. The results of this study may help to further explore the possibility of developing strategies to neutralize ANG2 in the treatment of nAMD.

## Additional Information

**How to cite this article:** Ng, D. S. *et al*. Elevated angiopoietin 2 in aqueous of patients with neovascular age related macular degeneration correlates with disease severity at presentation. *Sci. Rep.* **7**, 45081; doi: 10.1038/srep45081 (2017).

**Publisher's note:** Springer Nature remains neutral with regard to jurisdictional claims in published maps and institutional affiliations.

## Figures and Tables

**Table 1:** Demographics of patients in this study.

	nAMD	Control	P values
Number of patients	24	26	N/A
Age (mean +/− SD and range in years)	72.3 +/− 11.4, 60–87	73.7 + /− 14.5, 63–93	0.53
Number of patients with hypertension	15	17	0.37
Number of patients with hyperlipidemia	10	9	0.42
Number of patients with ischemic heart disease	6	6	0.21
CMT on OCT (mean +/− SD in μm)	353 +/− 140.34	229.05 +/− 26.99	<0.001
Lesion types	15 occult	N/A	N/A
	4 classic		
	6 min classic		

Central macular thickness (CMT) on optical coherence tomography (OCT) and lesion types on fluorescein angiography in patients with nAMD.

**Table 2:** Aqueous humour concentrations (pg/ml) of cytokines (mean, median and intrequartile range) in patients with nAMD and controls.

	nAMD mean, median, IQR (pg/l)	Control mean, median, IQR (pg/l)	Statistical difference between nAMD and Control group (P value)
VEGF	23.49, 21.90, 38.11	10.3, 8.25, 6.82	0.085
ANG2	57.39, 42.88, 48.86	10.46, 9.44, 8.10	<0.0001
FGF	25.7, 17.48, 0.00	21.18, 17.79, 5.60	0.139
HGF	333.3, 293.98, 121.79	28.11, 24.84, 10.87	<0.001
IL8	27.29, 28.83, 15.55	2.21, 2.10, 0.48	<0.001
TIMP1	24675.85, 24985.96, 9584.47	893.32, 864.18, 408.34	<0.001
TIMP2	15067.2, 17189.65, 25794.14	1112.03, 1011.09, 390.28	0.086
TNFα	5.89, 2.33, 3.67	8.49, 8.15, 9.98	0.224

Statistical significant differences can be found in Ang2, HGF, IL8 and TIMP1.

**Table 3:** Correlation coefficients of cytokines with BCVA and CMT on OCT.

	Correlation coefficient with BCVA	P value	Correlation coefficient with CMT on OCT	P value
VEGF	0.05	0.8	0.04	0.85
ANG2	0.44	0.027	0.66	<0.0001
FGF	0.31	0.14	0.49	0.012
HGF	0.19	0.37	0.14	0.51
IL8	0.002	0.99	0.15	0.484
TIMP1	0.04	0.86	0.03	0.877
TIMP2	0.29	0.17	0.06	0.77
TNFα	0.26	0.21	0.05	0.809

Only Ang2 is correlated with BCVA and CMT on OCT at presentation.
